# *rac*-(2a*S*,2a^1^ *R*,3a*R*,3a^1^ *S*,5a*S*,6a*R*)-2a-Allyl-2,4-di­chloro-2a,2a^1^,3a^1^,5a,6,6a-hexa­hydro-3a*H*-3-oxadi­cyclo­penta­[*cd*,*gh*]penta­len-3a-ol

**DOI:** 10.1107/S2414314621012608

**Published:** 2021-12-16

**Authors:** Sambasivarao Kotha, Saima Ansari, Naveen Kumar Gupta

**Affiliations:** a Department of Chemistry, Indian Institute of Technology Bombay, Powai, Mumbai - 400076, India; University of Aberdeen, Scotland

**Keywords:** crystal structure, triquinane, hemiketal, indium-catalysed, transannular cyclization

## Abstract

The title racemic triquinane, C_14_H_14_Cl_2_O_2_, is composed of four five-membered rings, one of which is a tetra­hydro­furan ring to which an allyl group on one side and a hydroxyl group on the other side are attached. The core of the triquinane unit has a *cis–syn–cis* configuration. In the crystal, the mol­ecules are linked by pairwise O—H⋯O hydrogen bonds, generating inversion dimers featuring *R* ^2^_2_(8) loops.

## Abstract 2

The title di­chloro-substituted hemiketal derivative of a *cis–syn–cis* triquinane is described.

## Graphical abstract

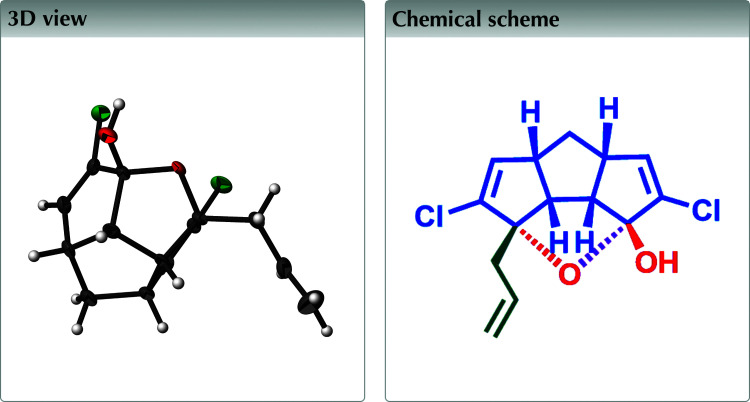

## Structure description

Compounds with three fused five-membered rings, known as triquinanes, have gained considerable importance because this core is found in several biologically active compounds (Qiu *et al.*, 2018 ▸; Kotha *et al.*, 2020 ▸). Therefore, convenient methods to prepare and functionalize triquinanes and the study of their stereochemistry are useful exercises (Mehta & Rao, 1985 ▸). Our group has prepared triquinanes from cage compounds in a simplified manner using microwave irradiation (Kotha *et al.*, 2019 ▸). Thereafter, we attempted to functionalize the triquinanes and observed a transannular attack at the keto centre (O1—C1—O2) leading to the formation of the title compound, **1**.

**Figure 1:**
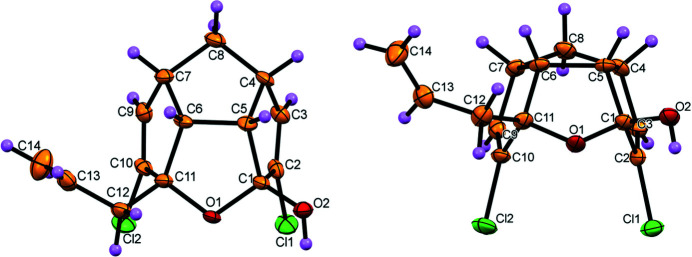
The mol­ecular structure of **1** (*a*) viewed from above and (*b*) viewed from the front. Displacement ellipsoids are drawn at the 50% probability level.

Compound **1** has three carbocyclic rings (C1/C2/C3/C4/C5, C4/C5/C6/C7/C8 and C6/C7/C9/C10/C11) and a tera­hydro­furan ring (O1/C1/C5/C6/C11). The allyl group is unsymmetrically substituted at C11 and the hy­droxy group is attached to C1 (Fig. 1 ▸ *a*). There are six stereogenic centres in **1**: in the arbitrarily chosen asymmetric mol­ecule, the configurations are C1 *R*, C4 *R*, C5 *S*, C6 *R*, C7 *S* and C11 *S* but crystal symmetry generates a racemic mixture.

The triquinane ring system consists of a *cis–syn–cis* configuration, *i.e*., the hydrogen atoms at the ring junction are all above the plane and the first and the third rings are below the plane (Fig. 1 ▸ *b*). The chlorine atoms are attached to the unsaturated bonds C2—C3 and C9—C10 in *anti*-manner with respect to the H atoms of the ring junction. The middle cyclo­pentyl ring adopts an envelope conformation and the side rings are almost planar.

**Figure 2:**
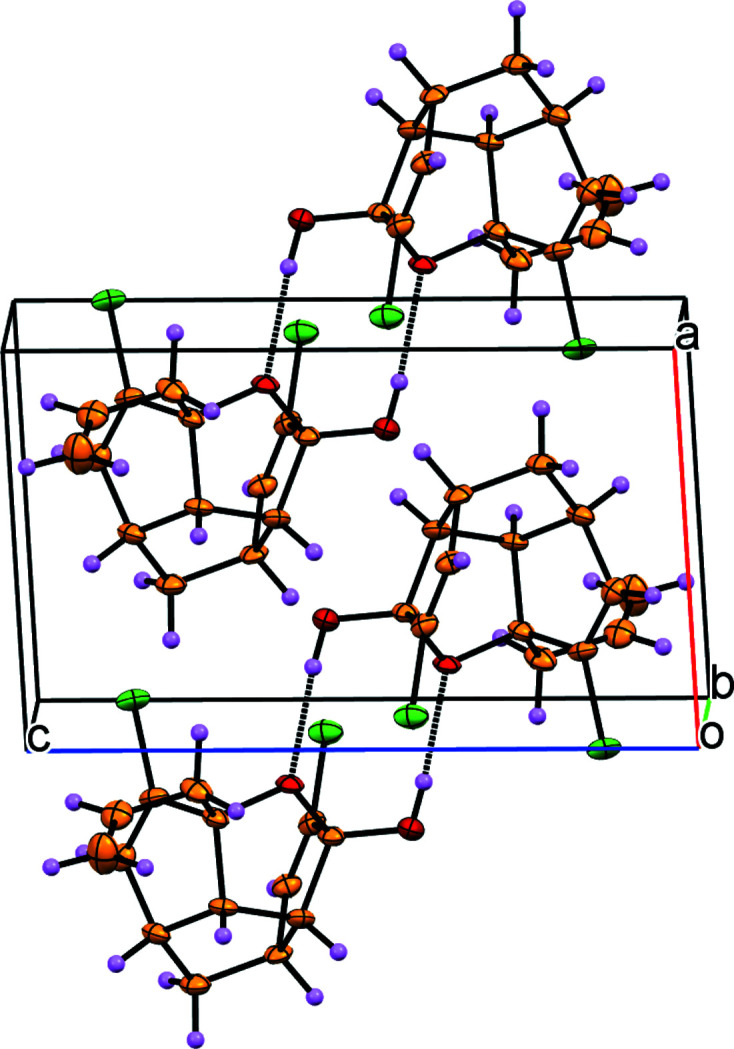
The crystal packing of **1**, viewed along the *b-*axis direction. The hydrogen bonding is shown using dotted lines.

In the crystal, the mol­ecules are linked by O—H⋯O hydrogen bonds, generating inversion dimers featuring 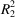 (8) loops (Table 1 ▸, Fig. 2 ▸) but no intra­molecular hydrogen bonds are present.

## Synthesis and crystallization

**Figure 3:**
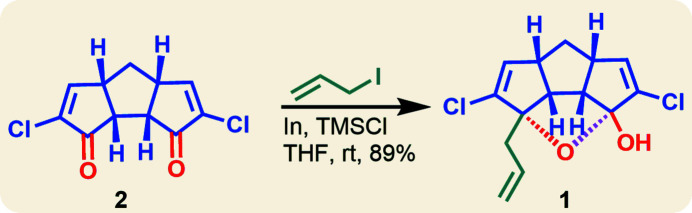
Synthesis scheme for **1**.

The synthesis scheme is shown in Fig. 3 ▸. Indium ingots (51 mg, 2.7 eq) were cut into small pieces and transferred to a two-neck round-bottomed flask. Tetra­hydro­furan (3 ml) was transferred to the flask under nitro­gen at room temperature. Allyl iodide (0.5 ml) was added to this solution *via* a syringe. After one h, the starting material **2** (40 mg) and tri­methyl­chloro­silane (3 drops) was added to the reaction mixture. On completion of the reaction (TLC monitoring) after 1 h, water was added to the reaction mixture. The aqueous layer was extracted with diethyl ether (Lee *et al.* 2001 ▸). The compound was purified with column chromatography and silica gel (100–200 mesh) was used. Ethyl acetate:petroleum ether (8% of ethyl acetate in total in 100 ml of solution) was used an eluent. After that, the crystals suitable for X-ray crystallographic analysis were grown in air in a glass vial using ethyl acetate as solvent (Fig. 3 ▸).

Characterization: colourless crystalline solid; m.p. 120–122°C; ^1^H NMR (500 MHz, CDCl_3_): *δ* = 5.73–5.62 (*m*, 3H), 5.20–5.14 (*m*, 2H), 3.39–3.30 (*m*, 2H), 3.23–3.20 (*m*, 1H), 3.02–2.98 (*m*, 1H), 2.63 (*dd*, *J* = 13.8, 7.0 Hz, 1H), 2.55 (*dd*, *J* = 13.8, 7.0 Hz, 1H), 1.95–1.87 (*m*, 1H), 1.78 (*d*, *J* = 13.9 Hz, 1H) p.p.m.; ^13^C NMR (125 MHz, CDCl_3_): *δ* = 134.1, 133.2, 133.1, 133.0, 132.5, 119.1, 115.5, 97.7, 58.8, 54.8, 47.7, 46.4, 40.4, 35.2 p.p.m.; HRMS (ESI): *m*/*z* calculated for C_14_H_14_Cl_2_NaO_2_ [*M* + Na]^+^: 307.0262; found: 307.0263.

## Refinement

Crystal data, data collection and structure refinement details are summarized in Table 2 ▸.

## Supplementary Material

Crystal structure: contains datablock(s) I. DOI: 10.1107/S2414314621012608/hb4394sup1.cif

Structure factors: contains datablock(s) I. DOI: 10.1107/S2414314621012608/hb4394Isup2.hkl

CCDC reference: 2114309

Additional supporting information:  crystallographic information; 3D view; checkCIF report

## Figures and Tables

**Table 1:** Hydrogen-bond geometry (Å, °)

*D*—H⋯*A*	*D*—H	H⋯*A*	*D*⋯*A*	*D*—H⋯*A*
O2—H2⋯O1^i^	0.84	2.06	2.893 (3)	173

Symmetry code: (i) 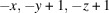 .

**Table 2:** Experimental details

Crystal data	
Chemical formula	C_14_H_14_Cl_2_O_2_
*M*_r_	285.15
Crystal system, space group	Triclinic, *P* 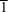
Temperature (K)	150
*a*, *b*, *c* (Å)	7.2687 (10), 8.3648 (11), 11.7460 (18)
α, β, γ (°)	80.448 (4), 83.441 (4), 65.285 (4)
*V* (Å^3^)	638.96 (16)
*Z*	2
Radiation type	Mo *K*α
μ (mm^−1^)	0.50
Crystal size (mm)	0.32 × 0.29 × 0.09

Data collection	
Diffractometer	Bruker APEXII CCD
Absorption correction	Multi-scan (*SADABS*; Bruker, 2016 ▸)
*T*_min_, *T*_max_	0.655, 0.746
No. of measured, independent and observed [*I* > 2σ(*I*)] reflections	19764, 2241, 1662
*R*_int_	0.106
(sin θ/λ)_max_ (Å^−1^)	0.594

Refinement	
*R*[*F* ^2^ > 2σ(*F* ^2^)], *wR*(*F* ^2^), *S*	0.048, 0.107, 1.09
No. of reflections	2241
No. of parameters	164
H-atom treatment	H-atom parameters constrained
Δρ_max_, Δρ_min_ (e Å^−3^)	0.27, −0.33

Computer programs: *APEX2* and *SAINT* (Bruker, 2016 ▸), *SHELXT* (Sheldrick, 2015*a* ▸), *SHELXL* (Sheldrick, 2015*b* ▸) and *OLEX2* (Dolomanov *et al.*, 2009 ▸).

## full crystallographic data

### Crystal data

**Table:**

C_14_H_14_Cl_2_O_2_	*Z* = 2
*M**_r_* = 285.15	*F*(000) = 296
Triclinic, *P*1	*D*_x_ = 1.482 Mg m^−^^3^
*a* = 7.2687 (10) Å	Mo *K*α radiation, λ = 0.71073 Å
*b* = 8.3648 (11) Å	Cell parameters from 3239 reflections
*c* = 11.7460 (18) Å	θ = 2.7–25.0°
α = 80.448 (4)°	µ = 0.50 mm^−^^1^
β = 83.441 (4)°	*T* = 150 K
γ = 65.285 (4)°	Plate, clear light colourless
*V* = 638.96 (16) Å^3^	0.32 × 0.29 × 0.09 mm

### Data collection

**Table:**

Bruker APEXII CCD diffractometer	1662 reflections with *I* > 2σ(*I*)
φ and ω scans	*R*_int_ = 0.106
Absorption correction: multi-scan (SADABS; Bruker, 2016)	θ_max_ = 25.0°, θ_min_ = 2.7°
*T*_min_ = 0.655, *T*_max_ = 0.746	*h* = −8→8
19764 measured reflections	*k* = −9→9
2241 independent reflections	*l* = −13→13

### Refinement

**Table:**

Refinement on *F*^2^	Primary atom site location: dual
Least-squares matrix: full	Hydrogen site location: inferred from neighbouring sites
*R*[*F*^2^ > 2σ(*F*^2^)] = 0.048	H-atom parameters constrained
*wR*(*F*^2^) = 0.107	*w* = 1/[σ^2^(*F*_o_^2^) + (0.0235*P*)^2^ + 1.1376*P*] where *P* = (*F*_o_^2^ + 2*F*_c_^2^)/3
*S* = 1.09	(Δ/σ)_max_ < 0.001
2241 reflections	Δρ_max_ = 0.27 e Å^−^^3^
164 parameters	Δρ_min_ = −0.33 e Å^−^^3^
0 restraints	

### Special details

**Table:**

Geometry. All esds (except the esd in the dihedral angle between two l.s. planes) are estimated using the full covariance matrix. The cell esds are taken into account individually in the estimation of esds in distances, angles and torsion angles; correlations between esds in cell parameters are only used when they are defined by crystal symmetry. An approximate (isotropic) treatment of cell esds is used for estimating esds involving l.s. planes.

### Fractional atomic coordinates and isotropic or equivalent isotropic displacement parameters (Å^2^)

**Table:**

	*x*	*y*	*z*	*U*_iso_*/*U*_eq_	
Cl1	0.06076 (13)	0.17026 (12)	0.42861 (8)	0.0269 (3)	
Cl2	−0.06759 (13)	0.53279 (13)	0.15245 (8)	0.0328 (3)	
O1	0.1461 (3)	0.5253 (3)	0.37821 (19)	0.0191 (5)	
O2	0.2783 (3)	0.3694 (3)	0.55085 (19)	0.0221 (6)	
H2	0.157708	0.391949	0.574798	0.033*	
C1	0.2966 (5)	0.3622 (4)	0.4322 (3)	0.0185 (8)	
C11	0.2225 (5)	0.5758 (4)	0.2653 (3)	0.0192 (8)	
C3	0.4551 (5)	0.0894 (4)	0.3551 (3)	0.0209 (8)	
H3	0.474728	−0.022512	0.335419	0.025*	
C10	0.1821 (5)	0.4890 (4)	0.1739 (3)	0.0194 (8)	
C6	0.4571 (5)	0.4848 (4)	0.2673 (3)	0.0179 (7)	
H6	0.518199	0.572267	0.266037	0.021*	
C2	0.2846 (5)	0.1995 (4)	0.4009 (3)	0.0198 (8)	
C9	0.3446 (5)	0.3828 (4)	0.1197 (3)	0.0211 (8)	
H9	0.342144	0.317963	0.061141	0.025*	
C4	0.6154 (5)	0.1623 (4)	0.3377 (3)	0.0203 (8)	
H4	0.732882	0.086130	0.386498	0.024*	
C7	0.5363 (5)	0.3772 (4)	0.1626 (3)	0.0206 (8)	
H7	0.604311	0.435233	0.101481	0.025*	
C8	0.6874 (5)	0.1927 (5)	0.2120 (3)	0.0240 (8)	
H8A	0.688215	0.100747	0.168389	0.029*	
H8B	0.826068	0.188696	0.207430	0.029*	
C5	0.5042 (5)	0.3485 (4)	0.3779 (3)	0.0195 (8)	
H5	0.584893	0.371253	0.431830	0.023*	
C13	0.1802 (5)	0.8510 (5)	0.1251 (3)	0.0270 (9)	
H13	0.152010	0.810452	0.061014	0.032*	
C12	0.1281 (5)	0.7775 (4)	0.2438 (3)	0.0253 (8)	
H12A	−0.021149	0.820022	0.254500	0.030*	
H12B	0.174476	0.823785	0.301924	0.030*	
C14	0.2611 (6)	0.9658 (5)	0.1035 (4)	0.0399 (11)	
H14A	0.291514	1.009520	0.165285	0.048*	
H14B	0.289666	1.006095	0.025767	0.048*	

### Atomic displacement parameters (Å^2^)

**Table:**

	*U*^11^	*U*^22^	*U*^33^	*U*^12^	*U*^13^	*U*^23^
Cl1	0.0202 (5)	0.0279 (5)	0.0360 (6)	−0.0142 (4)	−0.0005 (4)	−0.0023 (4)
Cl2	0.0145 (5)	0.0479 (6)	0.0353 (6)	−0.0122 (4)	−0.0051 (4)	−0.0026 (5)
O1	0.0093 (12)	0.0173 (12)	0.0240 (14)	0.0000 (10)	0.0021 (10)	−0.0013 (10)
O2	0.0154 (13)	0.0278 (14)	0.0207 (14)	−0.0065 (11)	0.0014 (10)	−0.0045 (11)
C1	0.0109 (17)	0.0171 (18)	0.023 (2)	−0.0020 (14)	0.0003 (14)	−0.0012 (15)
C11	0.0128 (18)	0.0183 (18)	0.022 (2)	−0.0031 (15)	0.0037 (14)	−0.0039 (15)
C3	0.0184 (19)	0.0157 (18)	0.027 (2)	−0.0049 (15)	−0.0040 (15)	−0.0026 (15)
C10	0.0112 (18)	0.0185 (18)	0.028 (2)	−0.0057 (15)	−0.0039 (15)	0.0008 (15)
C6	0.0095 (17)	0.0166 (18)	0.026 (2)	−0.0038 (14)	0.0024 (14)	−0.0049 (15)
C2	0.0189 (19)	0.0156 (18)	0.024 (2)	−0.0074 (16)	−0.0053 (15)	0.0039 (15)
C9	0.023 (2)	0.0166 (18)	0.022 (2)	−0.0073 (16)	−0.0035 (16)	−0.0008 (15)
C4	0.0108 (18)	0.0173 (18)	0.026 (2)	0.0015 (15)	−0.0040 (14)	−0.0026 (15)
C7	0.0134 (18)	0.0202 (18)	0.025 (2)	−0.0049 (15)	0.0038 (14)	−0.0021 (15)
C8	0.0125 (18)	0.024 (2)	0.030 (2)	−0.0022 (16)	−0.0001 (15)	−0.0045 (16)
C5	0.0116 (17)	0.0228 (19)	0.025 (2)	−0.0075 (15)	−0.0002 (14)	−0.0042 (15)
C13	0.026 (2)	0.0174 (19)	0.032 (2)	−0.0030 (16)	−0.0035 (16)	−0.0018 (16)
C12	0.019 (2)	0.0178 (19)	0.031 (2)	−0.0016 (16)	0.0050 (16)	−0.0032 (16)
C14	0.056 (3)	0.043 (3)	0.032 (2)	−0.033 (2)	−0.002 (2)	−0.0005 (19)

### Geometric parameters (Å, º)

**Table:**

Cl1—C2	1.731 (3)	C3—C4	1.506 (5)
Cl2—C10	1.734 (3)	C10—C9	1.315 (5)
O1—C1	1.444 (4)	C6—C7	1.557 (5)
O1—C11	1.445 (4)	C6—C5	1.545 (5)
O2—C1	1.394 (4)	C9—C7	1.515 (5)
C1—C2	1.506 (5)	C4—C8	1.526 (5)
C1—C5	1.536 (4)	C4—C5	1.552 (5)
C11—C10	1.508 (5)	C7—C8	1.534 (5)
C11—C6	1.550 (4)	C13—C12	1.501 (5)
C11—C12	1.520 (5)	C13—C14	1.300 (5)
C3—C2	1.316 (5)		
			
C1—O1—C11	110.0 (2)	C5—C6—C11	105.6 (3)
O1—C1—C2	112.9 (3)	C5—C6—C7	106.9 (3)
O1—C1—C5	107.0 (3)	C1—C2—Cl1	119.8 (2)
O2—C1—O1	107.8 (3)	C3—C2—Cl1	126.3 (3)
O2—C1—C2	113.5 (3)	C3—C2—C1	113.9 (3)
O2—C1—C5	113.0 (3)	C10—C9—C7	111.3 (3)
C2—C1—C5	102.5 (3)	C3—C4—C8	114.6 (3)
O1—C11—C10	111.4 (3)	C3—C4—C5	103.4 (3)
O1—C11—C6	106.4 (2)	C8—C4—C5	106.3 (3)
O1—C11—C12	106.8 (3)	C9—C7—C6	103.2 (3)
C10—C11—C6	101.7 (3)	C9—C7—C8	115.4 (3)
C10—C11—C12	113.9 (3)	C8—C7—C6	105.4 (3)
C12—C11—C6	116.5 (3)	C4—C8—C7	105.9 (3)
C2—C3—C4	111.8 (3)	C1—C5—C6	105.2 (3)
C11—C10—Cl2	118.3 (2)	C1—C5—C4	107.3 (3)
C9—C10—Cl2	126.5 (3)	C6—C5—C4	106.6 (3)
C9—C10—C11	115.1 (3)	C14—C13—C12	125.0 (4)
C11—C6—C7	107.5 (3)	C13—C12—C11	113.3 (3)

### Hydrogen-bond geometry (Å, º)

**Table:**

*D*—H···*A*	*D*—H	H···*A*	*D*···*A*	*D*—H···*A*
O2—H2···O1^i^	0.84	2.06	2.893 (3)	173

Symmetry code: (i) −*x*, −*y*+1, −*z*+1.
